# Sunitinib induced hepatotoxicity in L02 cells *via* ROS-MAPKs signaling pathway

**DOI:** 10.3389/fphar.2022.1002142

**Published:** 2022-10-26

**Authors:** Ting-Li Tang, Yan Yang, Lin Guo, Shuang Xia, Bikui Zhang, Miao Yan

**Affiliations:** Department of Pharmacy, The Second Xiangya Hospital, Central South University, Changsha, China

**Keywords:** Sunitinib, hepatotoxicity, apoptosis, autophagy, oxidative stress, MAPK, glycyrrhetinic acid

## Abstract

Sunitinib is a multi-targeted tyrosine kinase inhibitor with remarkable anticancer activity, while hepatotoxicity is a potentially fatal adverse effect of its administration. The aim of this study was to elucidate the mechanism of hepatotoxicity induced by Sunitinib and the protective effect of glycyrrhetinic acid (GA). Sunitinib significantly reduced the survival of human normal hepatocytes (L02 cells), induced the increase of alanine aminotransferase (ALT), aspartate aminotransferase (AST) and lactate dehydrogenase (LDH). Chloroquine (CQ) and Z-VAD-FMK were applied to clarify the cell death patterns induced by Sunitinib. Sunitinib significantly induced L02 cells death by triggering apoptosis and autophagy acted as a self-defense mechanism to promote survival. Sunitinib exposure caused excessive ROS generation which activated mitogen-activated protein kinases (MAPKs) signaling. Mechanistically, SP600125 (JNK inhibitor) and SB203580 (p38 inhibitor) respectively blocked apoptosis and autophagy induced by Sunitinib. And inhibition of ROS by NAC pretreatment ameliorated the effect of Sunitinib on MAPKs phosphorylation. GA alleviated Sunitinib-induced cell damage by inhibiting apoptosis and autophagy. These results suggested ROS/MAPKs signaling pathway was responsible for Sunitinib-induced hepatotoxicity and GA could be a preventive strategy to alleviate liver injury caused by Sunitinib.

## Introduction

Sunitinib, as an oral-administrated and multi-targeted tyrosine-kinase inhibitor (TKI), with significant curative effect, is approved for the treatment of metastatic renal cell carcinoma, imatinib-resistant gastrointestinal stromal tumor and pancreatic neuroendocrine tumor (Goodman et al., 2007). However, it can lead to unexpected toxic effects, such as fatal hepatotoxicity (Mueller et al., 2008; Studentova et al., 2022). A recent meta-analysis of Sunitinib-related adverse events in metastatic renal cell carcinoma reported that liver enzymes increased in 40% of 5,658 patients (Ibrahim et al., 2013). Cases of fulminant liver failure in patients treated with Sunitinib have been reported (Mueller et al., 2008; Taran et al., 2009; Mermershtain et al., 2013). Moreover, a black-box warning about rare but life-threatening hepatotoxicity treated after Sunitinib treatment was issued by Food and Drug Administration (FDA) (Shi et al., 2020). The underlying mechanism of hepatotoxicity caused by Sunitinib is needed further exploration. Therefore, further studies on the mechanism of Sunitinib-induced hepatotoxicity are urgently needed to shed light on a novel strategy for the intervention.

The molecular processes underlying the pathogenesis of liver injury generally involves apoptosis and autophagy (Wang, 2015; Ueno and Komatsu, 2017), which can be equally observed in TKIs-induced hepatotoxicity (Paech et al., 2017; Zhang et al., 2018). Apoptosis, a highly organized and genetically controlled type of cell death, is considered to be a prominent pathological feature in Sunitinib-related liver injury (Paech et al., 2018). Autophagy, a highly conserved self-digestion process, essential for cell survival as well as in the regulation of cell death, Studies have found that there is a complex interaction between autophagy and apoptosis. They can be activated by a variety of stimuli at the same time, jointly determining the progression of liver disease like drug-induced liver injury (Wang, 2015). Our group previously found Sunitinib induced hepatotoxicity through apoptosis (Guo et al., 2021). However, whether autophagy participates in the regulation of Sunitinib-induced hepatotoxicity remains unknown, which prompts us to examine the occurrence of autophagy and the cross-talk between apoptosis and autophagy in hepatocyte. Mitogen-activated protein kinases (MAPKs), comprising a family of the extracellular signal-regulated kinase (ERK), p38 and c-Jun N-terminal kinase (JNK), are important signaling components which convert extracellular signals into a wide range of cellular responses including proliferation, survival, differentiation, apoptosis and autophagy (Webber, 2010; Arthur and Ley, 2013; Yue and Lopez, 2020). Among them, ERK is usually responsible for cell proliferation and differentiation, while JNK and p38 are implicated in apoptosis and autophagy evoked by a variety of physical, chemical, and biological stress stimuli (Zhang and Liu, 2002; McClung et al., 2010). It has been reported that autophagy protects against dasatinib-induced hepatotoxicity *via* oxidative stress-driven p38-MAPK signaling (Yang et al., 2015). However, it is unclear whether MAPK signaling is involved in Sunitinib-induced hepatotoxicity.

Glycyrrhetinic acid (GA) is the main active component of licorice and has been used in clinical treatments because of its anti-oxidant and anti-inflammatory activities (Li et al., 2019; Cao et al., 2020). It is reported that GA has hepatoprotective effects in several liver injury models, such as carbon tetrachloride-induced liver injury, azathioprine-induced hepatotoxicity and acetaminophen-induced liver damage (Wu et al., 2006; Chen et al., 2013; Yang et al., 2017). Additionally, we observe that Sunitinib is often used in combination with licorice in clinical, while the exact mechanisms are still unclear.

This study is a continuation of the previous one. We investigated the role of autophagy and the interaction between apoptosis and autophagy in Sunitinib-mediated hepatotoxicity and found ROS-driven JNK/p38-MAPK signaling pathway is the underlying mechanism. To our knowledge, this is the first time the protective mechanism of GA to Sunitinib-induced liver injury is reported.

## Materials and methods

### Reagents

Sunitinib (purity≥98%) was purchased from Huateng pharmaceuticals-company (Hunan, China) and was dissolved in dimethyl sulfoxide (DMSO) (50 mM stock solution) and stored at −20°C and kept away from light. GA (purity≥97%) and NAC were supplied from Xiya Reagent Co., Ltd. (Shandong, China) and Solarbio (Beijing, China), respectively. Z-VAD-FMK was purchased from Meilunbio. SP600125, SB203580, chloroquine (CQ), 3-methyl adenine (3-MA) and Rapamycin were purchase from Selleck Chemicals (Houston, United States).

### Cell culture

The L02 cells (purchased from Shanghai Zhong Qiao Xin Zhou Biotechnology Co., Ltd.) are human normal hepatocytes widely used for studying hepatic pathophysiology as an *in vitro* model of liver tissue including hepatotoxicity (Zhang et al., 2020; Zhu et al., 2021). Cells were cultured at 37°C with 5% CO_2_ in DMEM medium (Gibco, Grand Island, NY, United States) supplemented with 10% FBS (Biological Industries, Israel)+1% penicillin/streptomycin (Gibco, United States). In all experiments, the cells were plated at an appropriate density according to the experimental design.

### MTT assay

L02 cells (5 × 10^4^/ml) were seeded in 96-well plates. After treatment with varying concentrations of Sunitinib and/or other drugs for a certain time, cell viability was detected using the MTT (BIOFROXX, Germany) assay at 490 nm. Then the relative cell viability (%) was the ratio of the absorbance of the administration cells to that of the untreated cells.

### Measurement of liver enzymes

After L02 cells were treated with drugs, the supernatant was collected, the levels of ALT, AST and LDH were determined by the full-automatic clinical analyzer in the laboratory of the second xiangya hospital (7,600, HITACHI Ltd, Tokyo, Japan).

### Measurement of intracellular ROS

ROS were measured with Reactive Oxygen Species Assay Kit (S0033S, Beyotime Biotech, Shanghai, China) according to the manufacturer’s instructions. In brief, L02 cells were exposed to sunitinib at different concentrations and times, the cells then were incubated with H2DCFDA for 30 min at 37°C, and measured immediately by fluorescence microscope (Olympus, Japan). The fluorescence intensity was quantified by using ImageJ software 1.52a.

### Hochest 33,342 staining assay

Apoptosis was detected by Hoechst 33,342 staining (C1022, Beyotime Biotech, Shanghai, China). In brief, after the treated cells were washed twice by PBS, fixed with 4% paraformaldehyde for 30 min, then incubated at 37°C for additional 30 min. After washed by PBS, the cells were observed and photographed at excitation wavelength 346 nm (Ex) and emission wave length 460 nm (Em) using the fluorescence microscope. Quantification was performed by counting condensed/fragmented nuclei.

### Monodansylcadaverine Staining Assay.

MDC is a fluorescent pigment which is commonly used as a specific marker stain for the detection of autophagosome formation (Corral-Ramos et al., 2015; Luo et al., 2020). Cells were washed with PBS and incubated with 50 μmol/L MDC (30,432, Sigma) at 37°C for 60 min, and then fixed with 4% paraformaldehyde for 15–30 min. After being washed thrice by phosphate buffer saline (PBS), the cells were observed and photographed at Ex 355 nm and Em 512 nm using the fluorescence microscope (Olympus, Japan). The quantification was performed by using ImageJ software 1.52a.

### Transmission electron microscope

L02 cells were fixed with 2.5% glutaraldehyde, pre-embedded in 1% agar, post-fixed with 1% osmic acid, dehydrated, embedded, sectioned at a thickness of 60–80 nm. The slice was stained with uranyl acetate and lead citrate. The ultrastructure of the cells was observed and photographed under a transmission electron microscope (HITACHI, HT7800).

### Western blotting

The protein samples were extracted in enhanced RIPA lysis buffer (P0013B, Beyotime Biotech, Shanghai, China) and supplemented with a protease and phosphatase inhibitor cocktail (ThermoFisher, United States). The equal amounts of total proteins were loaded and electrophoresed on 10% Tris-glycine precast gels, transferred to PVDF membrane (Millipore, Bedford, MA), and then probed with primary antibodies overnight. Antibodies for PARP (bf9106, Affinity, 1:1,000), cleaved caspase3 (af7022, Affinity, 1:1,000), Nrf2 (sc-365949, Santa cruz1:800), P62 (#3868, Abmart, 1:1,000), HO-1 (10701-1-AP, Proteintech, 1:1,000), LC3B (#3868, Cell Signaling Technology, 1:1,000), Bax (ab32503, Abcom, 1:1,000), Bcl2 (ab692, Abcom, 1:800), p-JNK (#4668T, Cell Signaling Technology, 1:1,000), JNK (sc-7345, Santa cruz, 1:1,000), p-P38 (#4511T, Cell Signaling Technology, 1:1,000), P38 (sc-7972, Santa cruz, 1:1,000), p-ERK (ap1120, Abclonal, 1:1,000), ERK (67170-1-Ig, Proteintech, 1:1,000), *β*-actin (66009-1-Ig, Proteintech, 1:5,000), GAPDH (GB12002, Servicebio, 1:5,000) were purchased. Then the membrane was incubated for 1 h with goat-anti rabbit IgG horseradish peroxidase (HRP) secondary antibody (#HG-SAM00002b, HonorGene, 1:5,000) or goat-anti mouse IgG HRP secondary antibody (#HG-SAM00001b, HonorGene, 1:5,000). Finally, bands were detected with ECL kit (NCM Biotech, Suzhou, China) by a Chemidoc Imager (BIO-RAD, United States) and the intensity of bands were quantified by Image Lab software (version 6.0).

### Statistical analysis

Statistical analyses were performed using GraphPad prism 8 software and the data were presented as mean ± SD. The independent-sample *t*-test was used for the comparison of two groups of data, and one-way analysis of variance (ANOVA) was used for the comparison of three or more groups of data. When the data met the homogeneity of variance test, LSD test was used for paired comparison between groups, and Dunnett’s T3 test was used for non-homogeneity of variance. When *p* ≤ 0.05, the difference between groups was considered statistically significant.

## Results

### Apoptosis contributes to sunitinib-induced hepatotoxicity

We embarked on our study by validating the toxic effect of Sunitinib in L02 cells. L02 cells were treated with different concentrations of Sunitinib for 12, 24, 48 h and the cell viability were measured by performing MTT assays. As shown in Supplementary Figure S1A, Sunitinib damaged L02 cells in dose and time-dependent manner. In addition, we observed that the level of ALT, AST, and LDH increased significantly after cells were treated with 15 μM of Sunitinib for 48 h (Supplementary Figure S1B). These results indicated that Sunitinib could induce hepatotoxicity *in vitro*.

**FIGURE 1:**
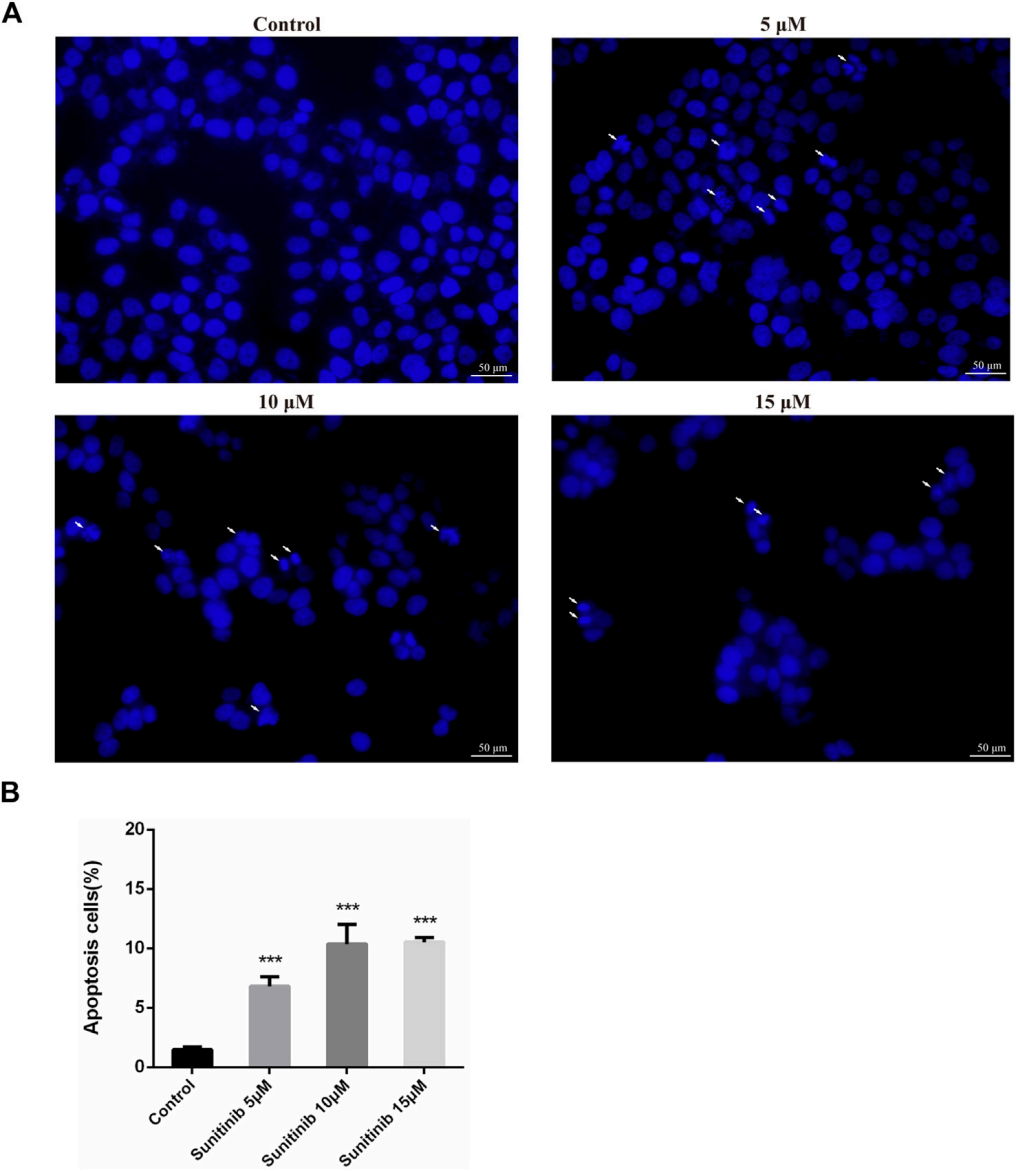
Sunitinib causes apoptosis in L02 cells. **(A)** Hepatocyte apoptosis was detected by Hoechst 33,342 staining in L02 cells. L02 cells were stained with Hoechst 33,342 after Sunitinib treatment at various concentration for 48 μh, and nuclear changes were observed on fluorescence microscopy (scale bars = 50 μm). Insets white arrows denote apoptosis hepatocytes (*n* = 3). **(B)** Quantification of apoptotic cells (%). ***p < 0.001 vs. Control group.

To evaluate whether Sunitinib-induced hepatotoxicity involves apoptosis, we stained L02 cells with Hoechst33342 to observe apoptotic morphological changes. Cells were pyknotic and the nucleus was pyknotic and hyperchromatic. Apoptotic cells were observed in Sunitinib-treated L02 cells (Figures 1A,B). Meanwhile, western blot analysis showed that the anti-apoptotic protein (Bcl-2) was downregulated and pro-apoptotic proteins (Bax) were found to be upregulated upon treatment with Sunitinib (Supplementary Figure S1C). And the protein levels of c-Caspase3 increased, and PARP decreased with the exposure of Sunitinib (Supplementary Figures S1D–E). Afterwards, we performed MTT assay to L02 cells after treatment with Sunitinib plus caspase inhibitor Z-VAD-FMK. The results demonstrated that Z-VAD-FMK pretreatment can increase the survival rate of L02 cells compared to Sunitinib-treated alone (Supplementary Figure S1F) and inhibit the increase of c-Caspase3 caused by Sunitinib (Supplementary Figures S1G,H). We thus concluded that Sunitinib induced cytotoxicity in the liver by promoting hepatocyte apoptosis.

### Autophagy protects against sunitinib-induced hepatotoxicity

**FIGURE 2:**
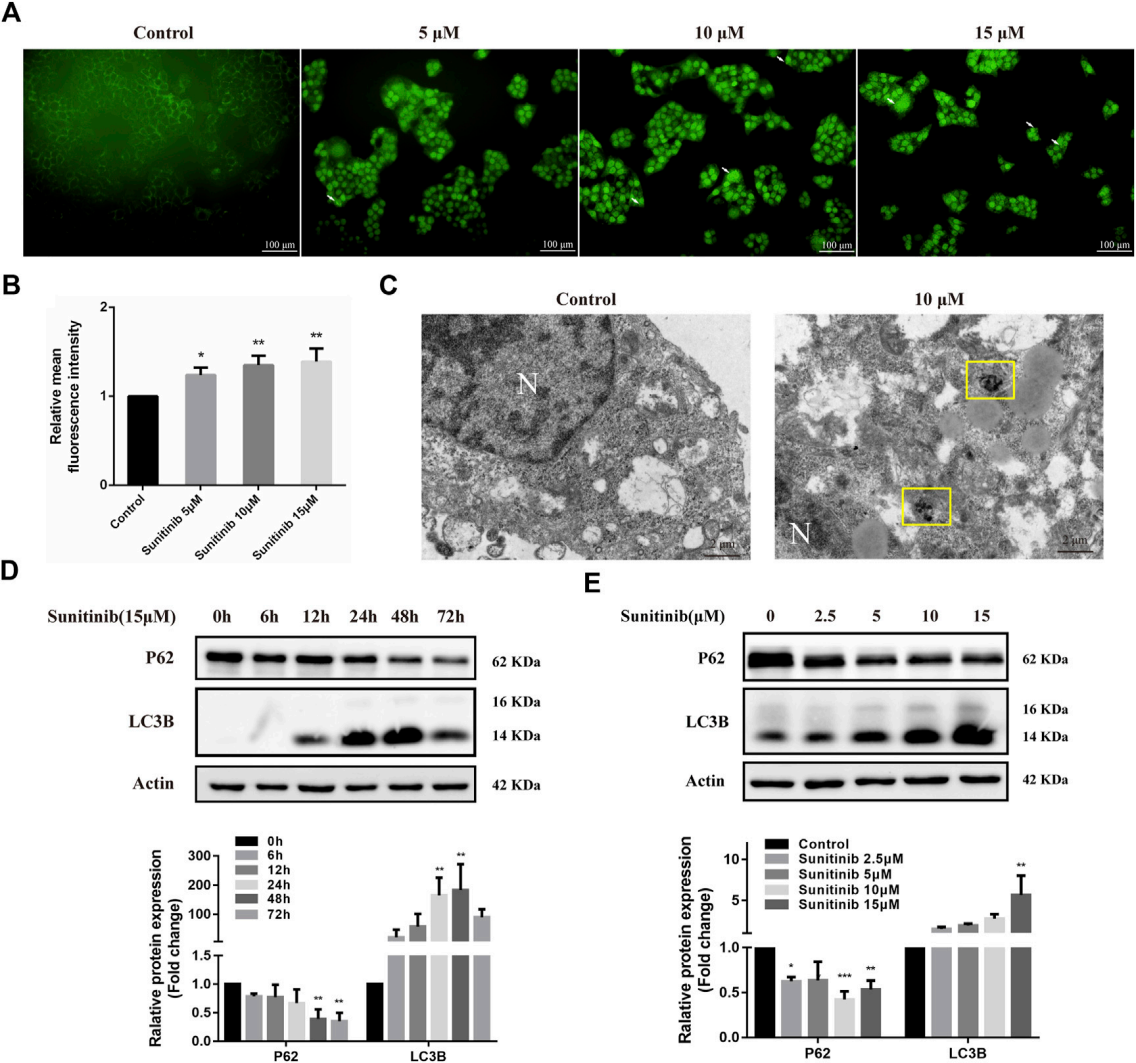
Sunitinib leads to autophagy. **(A)** Autophagic vacuoles were detected by MDC staining after Sunitinib exposure at 0, 5, 10, 15 μM for 48 h, and autophagosomes were observed on fluorescence microscopy (scale bars = 100 μm). White arrows denote autophagosomes. **(B)** Quantification of MDC intensities (*n* = 3). **(C)** Autophagosomes were detected by TEM after Sunitinib exposure at 0, 10 μM for 48 h (scale bars = 2 μm). Yellow squares denote autophagosomes. N, nuclear. **(D)** P62 and LC3B levels in L02 cells incubated with Sunitinib (15 μM) for 0, 6, 12, 24, 48, 72 h were measured (*n* = 3). **(E)** P62 and LC3B protein expression in L02 cells incubated with Sunitinib (0, 2.5, 5, 10, 15 μM) for 48 h were measured (*n* = 3). *p < 0.05, **p < 0.01 and ***p < 0.001 vs. Control group.

**FIGURE 3:**
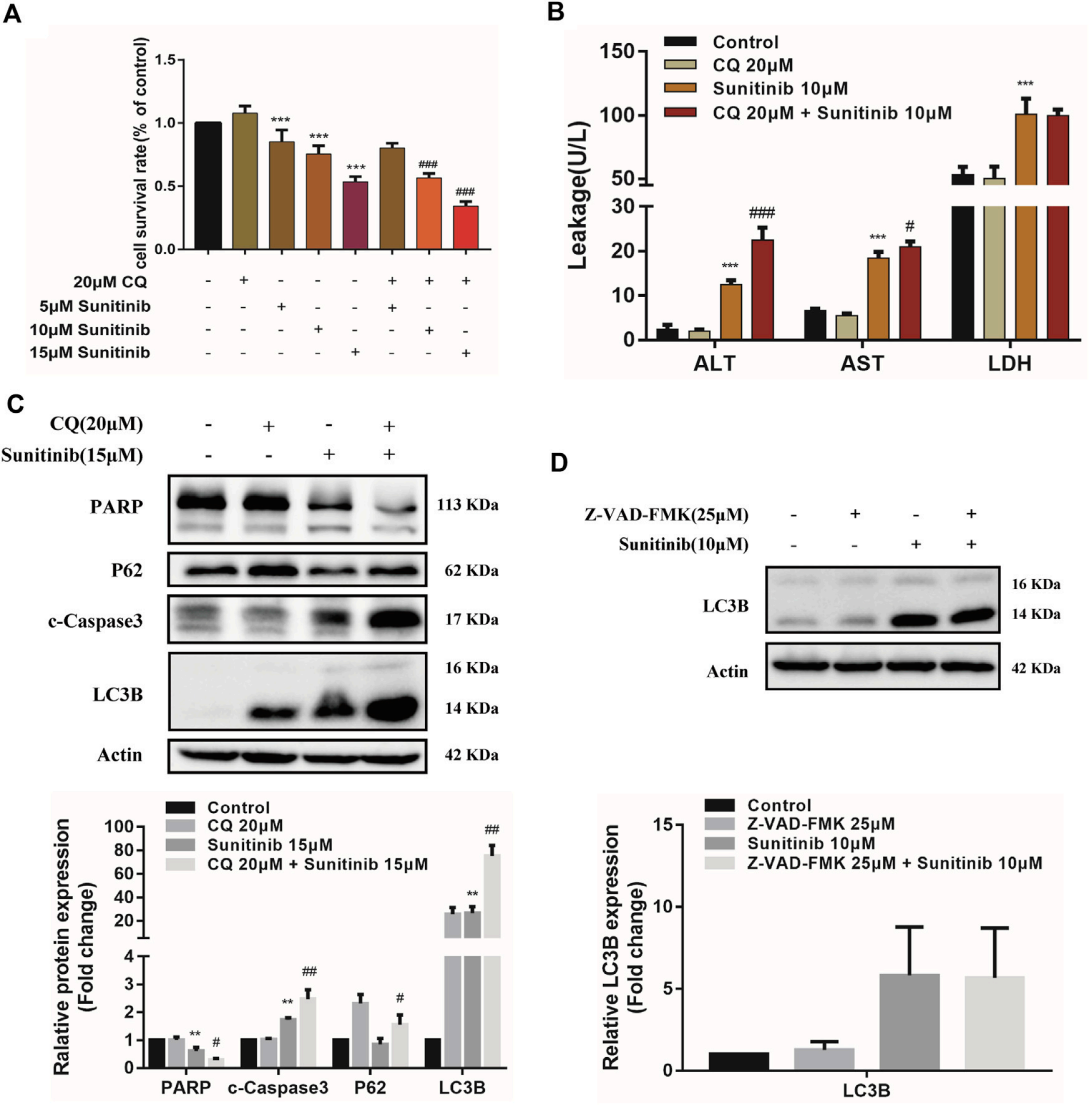
The interaction between apoptosis and autophagy. **(A)** The viability of hepatocytes was analyzed by MTT after L02 cells were incubated with 5, 10, 15 μM Sunitinib for 48 h in the presence or absence of 20 μM CQ pretreatment for 2 h (*n* = 6). **(B)** Levels of ALT, AST, and LDH released into the supernatant after L02 cells were incubated with 10 μM Sunitinib for 48 h in the presence or absence of 20 μM CQ pretreatment for 2 h (*n* = 3). **(C)** Protein expression of PARP, c-Caspase3, P62, and LC3B (*n* = 3). **(D)** Protein expression of LC3B after L02 cells were incubated with 10 μM Sunitinib for 48 h in the presence or absence of 25 μM Z-VAD-FMK pretreatment for 2 h (*n* = 3). ^**^ *p* < 0.01 and ^***^ *p* < 0.001 vs. Control group. ^#^ *p* < 0.05, ^##^ *p* < 0.01, and ^###^ *p* < 0.001 vs. Sunitinib group.

To determine whether Sunitinib induced autophagy, L02 cells were stained with MDC to detect autophagosome. As shown in Figures 2A,B, Sunitinib apparently promoted the formation of autophagic vesicles compared with the vehicle. Transmission electron microscopic observations, the gold standard for detecting autophagy, also revealed that Sunitinib treatment led to the accumulation of autophagic vacuoles in L02 cells (Figure 2C). Furthermore, considering LC3B is essential for the formation of the autophagosome and p62 is a protein degraded by autophagy, we detected the levels of LC3B and p62 by western blotting. It was found that exposure of L02 cells to Sunitinib resulted in an increase in endogenous LC3B-Ⅱ levels and degradation of p62 at 48 h (Figures 2D,E)., Next, we proceeded to examine the effect of autophagy on Sunitinib-induced hepatotoxicity. We found that combination of CQ (inhibitor of the autophagolysosomal degradation) at 20 μM decreased the survival of L02 cells (Figure 3A) and elicited evident increment of ALT with modest effect on AST levels and no effect on LDH levels compared to Sunitinib only treated group (Figure 3B). These results suggested that Sunitinib induced protective autophagy.

To further investigate the interaction between apoptosis and autophagy, we measured apoptosis level after the autophagy inhibitor CQ treatments. Inhibition of autophagy by CQ significantly increased the LC3B-Ⅱ accumulation and blocked p62 degradation, aggravated Sunitinib-induced elevation of cleaved-caspase-3 and decrease of PARP (Figure 3C). Apoptosis increased significantly in response to the inhibition of autophagy induced by Sunitinib. Then we examined the effect of apoptosis inhibition Z-VAD-FMK on autophagy. Z-VAD-FMK did not reverse the upregulation of LC3B-Ⅱ caused by Sunitinib (Figure 3D), which meant blocking apoptosis failed to prevent the Sunitinib-induced autophagy. Collectively, these results revealed that autophagy protected against apoptosis-related hepatotoxicity induced by Sunitinib.

### Sunitinib regulates apoptosis and autophagy *via* mitogen-activated protein kinases pathway

**FIGURE 4:**
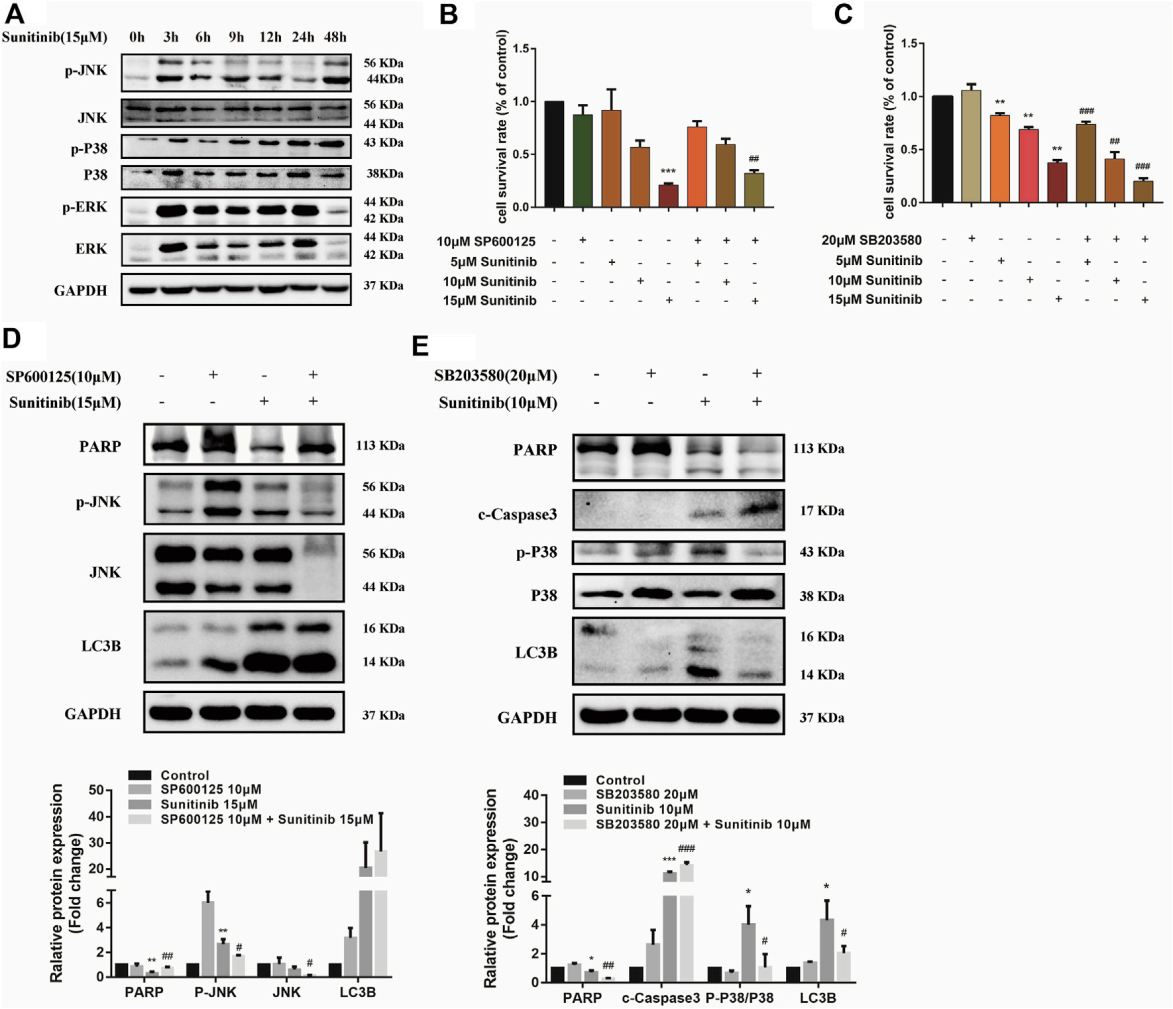
Sunitinib regulates apoptosis and autophagy *via* MAPKs pathway. **(A)** Detection proteins expression of JNK, P38, ERK and their phosphorylation incubated with Sunitinib (15 μM) for 0–48 h. **(B)** The viability of hepatocytes was analyzed by MTT after L02 cells were incubated with 5, 10, 15 μM Sunitinib for 24 h in the presence or absence of 10 μM SP600125 pretreatment for 2 h (*n* = 6). **(C)** The viability of hepatocytes was analyzed by MTT after L02 cells were incubated with 5, 10, 15 μM Sunitinib for 48 h in the presence or absence of 20 μM SB203580 pretreatment for 2 h (*n* = 6). **(D)** Protein expression of p-JNK, JNK, PARP, and LC3B after L02 cells were incubated with 15 μM Sunitinib for 24 h in the presence or absence of 10 μM SP600125 pretreatment for 2 h (*n* = 3). **(E)** Protein expression of p-P38, P38, PARP, c-Caspase3, and LC3B after L02 cells were incubated with 10 μM Sunitinib for 48 h in the presence or absence of 20 μM SB203580 pretreatment for 2 h (*n* = 3). ^*^ *p* < 0.05, ^**^ *p* < 0.01 and ^***^ *p* < 0.001 vs. Control group. ^#^ *p* < 0.05, ^##^ *p* < 0.01 and ^###^ *p* < 0.001 vs. Sunitinib group.

Considering MAPKs signaling is associated with various cellular stimuli and has been shown to contribute to regulation of apoptosis and autophagy, we were encouraged to examined the effects of Sunitinib on MAPKs signaling pathway. Our results indicate that Sunitinib induced a sharp increase in the phosphorylation of ERK, p38 and JNK after 3 h (Figure 4A). Then we combined JNK inhibitor (SP600125) and p38 inhibitor (SB203580) with Sunitinib to illustrate the role of JNK and p38 in Sunitinib-induced apoptosis and autophagy. It was found that JNK inhibitor SP600125 pretreatment was able to mitigate cell death (Figure 4B) and p38 inhibitor SB203580 pretreatment intensified cell death in Sunitinib-treated hepatocyte (Figure 4C). SP600125 reversed the reduction of PARP induced by Sunitinib, but did not affect the expression levels of LC3B (Figure 4D). It meant that Sunitinib might regulate apoptosis via JNK-MAPK pathway. Further results showed that inhibition of phosphorylation of p38 resulted in higher c-Caspase3 and lower LC3B-Ⅱ and PARP protein level (Figure 4E). In brief, p38 activation is an essential step for the stimulation of Sunitinib-mediated autophagy.

### ROS is responsible for activation of mitogen-activated protein kinases pathway

**FIGURE 5:**
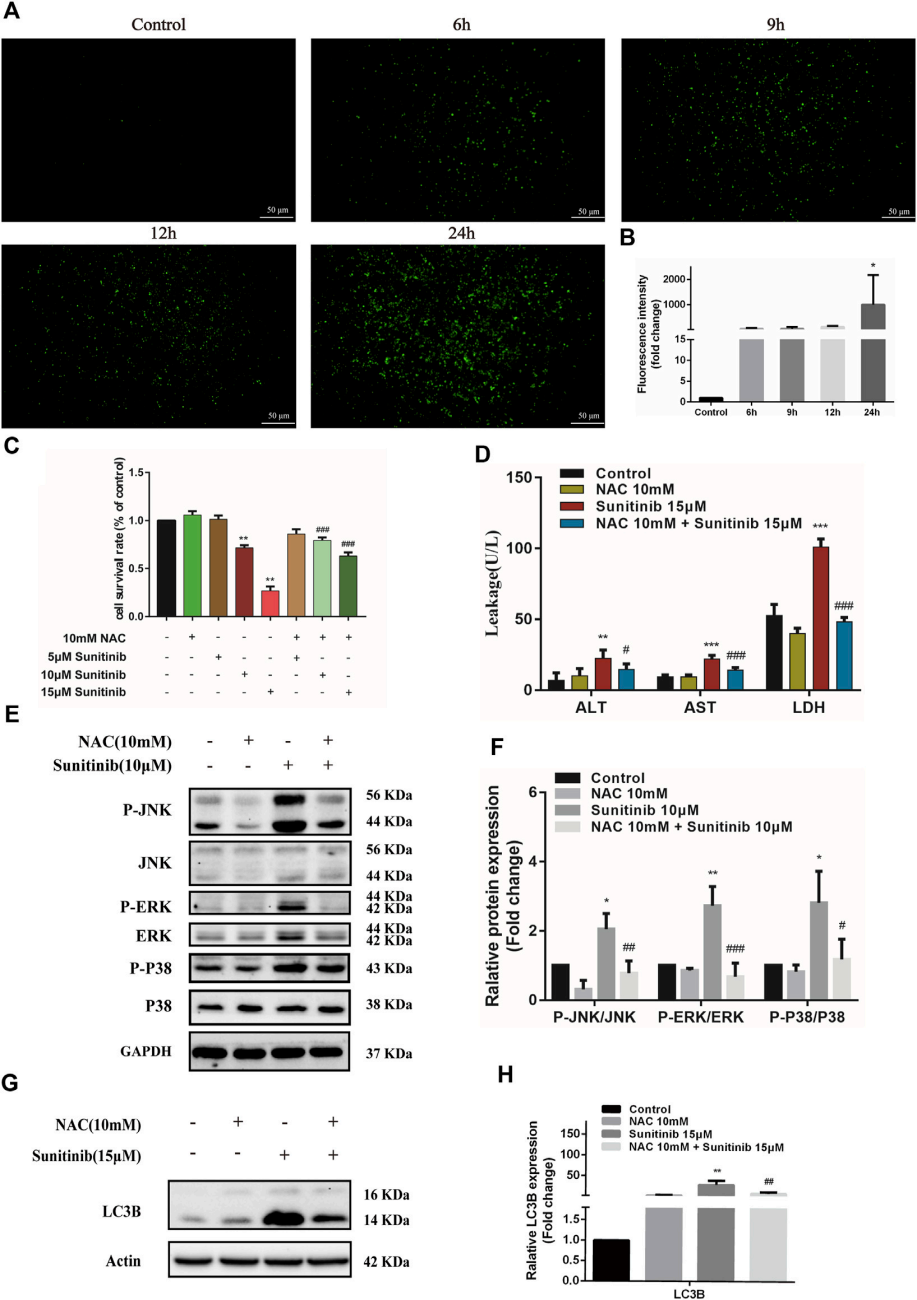
Sunitinib induces excessive ROS which activates MAPKs, thereby inducing apoptosis and autophagy in L02 cells. **(A)** L02 cells were exposed with Sunitinib at 15 μM for 6, 9, 12, 24 h. ROS levels were analyzed using a ROS assay kit. Green fluorescence was observed on fluorescence microscopy (scale bars = 50 μm). **(B)** Quantification of ROS intensities. **(C)** The viability of hepatocytes was analyzed by MTT after L02 cells were incubated with 5 μM, 10 μM, and 15 μM Sunitinib for 48 h in the presence or absence of 10 mM NAC pretreatment for 6 h (*n* = 6). **(D)** ALT, AST, and LDH levels in the supernatant after L02 cells were incubated with 15 μM Sunitinib for 48 h in the presence or absence of 10 mM NAC pretreatment for 6 h (*n* = 3). **(E,F)** Protein expression of p-JNK/JNK, p-ERK/ERK, and p-P38/P38 after L02 cells were incubated with 10 μM Sunitinib for 12 h in the presence or absence of 10 mM NAC pretreatment for 6 h (*n* = 3). **(G,H)** LC3B levels in L02 cells after Sunitinib (15 μM) for 48 h in the presence or absence of 10 mM NAC pretreatment for 6 h (*n* = 3). *p < 0.05, **p < 0.01, and ***p < 0.001 vs. Control group. ^#^ *p* < 0.05, ^##^ *p* < 0.01 and ^###^ *p* < 0.001 vs. Sunitinib group.

Oxidative stress is known to involve in the activation of MAPK and be responsible for the hepatotoxicity of many drugs. To evaluate whether Sunitinib triggered oxidative stress, L02 cells were treated with Sunitinib for various times (0–24 h) and incubated with H2DCFDA to reflects ROS level. We found that Sunitinib elicited the overproduction of ROS, with significant increment in H2DCFDA fluorescence (Figures 5A,B). Additionally, western blot analysis was employed to detect the expression of Nrf2 which is a key regulator against oxidative stress. We noticed that Sunitinib treatment markedly resulted in reduction of Nrf2 protein expression and its downstream target HO-1 (Supplementary Figures 2A,B). These results suggested that Sunitinib induced oxidative stress in hepatocytes. As we expected, NAC pretreatment, a scavenger of ROS, significantly improved the survival rate of L02 cells compared with Sunitinib treatment alone (Figure 5C), as well as decreased ALT, AST, and LDH levels (Figure 5D). To examine whether ROS mediated MAPK pathway, western blot analysis was employed and the results demonstrated that the phosphorylation of JNK, ERK, and p38 proteins were suppressed after NAC pretreatment (Figure 5E). Moreover, LC3B-Ⅱ accumulation, PARP and Nrf2 reduction was significantly attenuated due to NAC pretreatment (Figure 5G, Supplementary Figure S3). These findings indicated that ROS could activate MAPK signaling and play an important role in Sunitinib-induced hepatotoxicity.

### Glycyrrhetinic acid has protective effects against Sunitinib-induced hepatotoxicity

**FIGURE 6:**
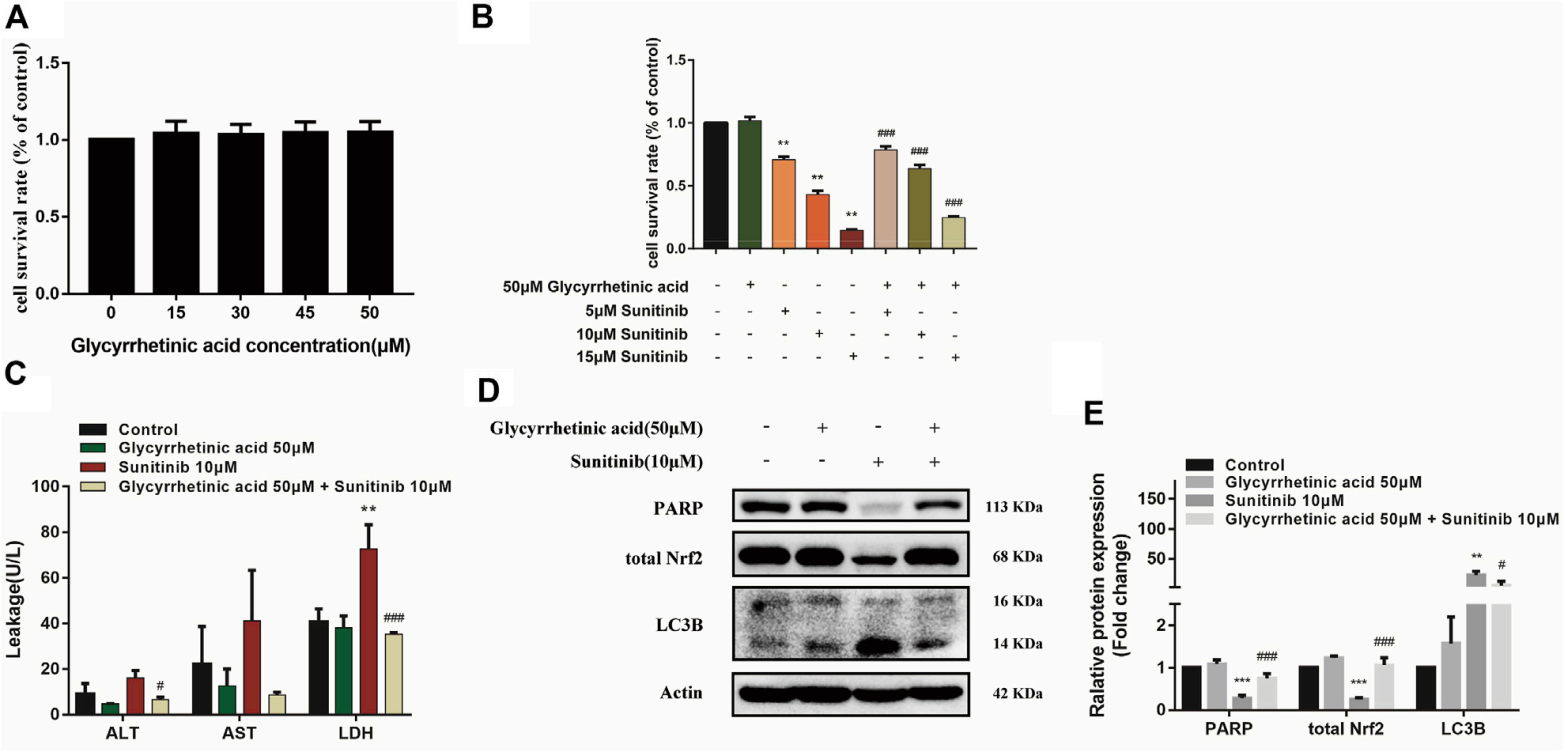
Glycyrrhetinic acid has protective effects against Sunitinib-induced hepatotoxicity. **(A)** Cytotoxicity of glycyrrhetinic acid at various concentration for 6 + 48 h in L02 cells (*n* = 4–6). **(B)** The viability of hepatocytes was analyzed by MTT after L02 cells were incubated with 5, 10, 15 μM Sunitinib for 48 h in the presence or absence of 50 μM glycyrrhetinic acid pretreatment for 6 h (*n* = 6). **(C)** ALT, AST and LDH levels in the supernatant after L02 cells were incubated with 10 μM Sunitinib for 48 h in the presence or absence of 50 μM glycyrrhetinic acid pretreatment for 6 h (*n* = 3). **(D,E)** LC3B levels in L02 cells after Sunitinib (10 μM) for 48 h in the presence or absence of 50 μM glycyrrhetinic acid pretreatment for 6 h (*n* = 3). **p < 0.01 and ***p < 0.001 vs. Control group. ^#^ *p* < 0.05, ^##^ *p* < 0.01 and ^###^ *p* < 0.001 vs. Sunitinib group.

Previous studies of our research group demonstrated that glycyrrhetinic acid exerted hepatic protective effects *via* antioxidant and antiapoptotic (Cao et al., 2016; Yan et al., 2021). Thus, in this study, we further investigated the effect of glycyrrhetinic acid on Sunitinib-induced hepatotoxicity. It was shown that glycyrrhetinic acid alone did not significantly affect cell viability at the concentration range of 0–50 μM (Figure 6A). Pretreatment with 50 μM glycyrrhetinic acid significantly reversed the decrease in cell viability (Figure 6B) and the increase in liver enzymes (ALT, AST, LDH) induced by Sunitinib (Figure 6C). And pretreatment with glycyrrhetinic acid could significantly restore the level of un-cleaved PARP, as well as inhibit the accumulation of LC3B-Ⅱ (Figures 6D,E). Furthermore, glycyrrhetinic acid obviously rescued Sunitinib-induced down-regulation of Nrf2 protein (Figures 6D,E). These findings reflected that glycyrrhetinic acid could protects against Sunitinib-induced hepatotoxicity and the possible mechanism is associated with alleviation of oxidative stress, reduction of apoptosis and autophagy.

## Discussion

Studies have found that Sunitinib can effectively treat many solid tumors (Goodman et al., 2007; Speed et al., 2012). However, hepatotoxicity of Sunitinib which ranges from common elevated liver enzymes to rare but lethal hepatic failure limits its application value (Guillen et al., 2016; Studentova et al., 2022). At present, there is no better intervention measure for liver toxicity caused by Sunitinib except reducing the dosage or stopping the medication. Reduction and discontinuation inevitably lead to treatment failure and cancer progression (Xiao et al., 2019). Therefore, studying underlying mechanisms of Sunitinib-induced hepatotoxicity and finding the corresponding intervention on this basis are of great significance to provide safety to patients.

Sunitinib has been shown to produce cytotoxic effects on HepG2 cells, HepaRG cells, rat and human primary hepatocytes (Paech et al., 2017; Zhang et al., 2018). Sunitinib could damage the mitochondrial electron transport chain resulting in hepatocyte apoptosis (Paech et al., 2018). We also proposed in our previous study that Sunitinib-induced hepatotoxicity was associated with apoptosis, mainly focusing on the mechanism of mitochondrial damage (Guo et al., 2021; Guo et al., 2022). In this study, changes in expression of apoptosis-related proteins were observed, with c-Caspase3 and Bax upregulated, PARP and Bcl2 down-regulated. Inhibiting apoptosis with Z-VAD-FMK resulted in alleviation of Sunitinib-triggered cell death. These data corroborated our conclusion that Sunitinib led to liver damage by promoting hepatocyte apoptosis. Due to the interference of the bright yellow color of Sunitinib, Annexin V/PI double staining combined with flow cytometry could not be used to determine the apoptosis of hepatocytes induced by Sunitinib. In this study, we first conducted MTT assay to investigate the damage of Sunitinib on human normal hepatocytes L02. The results showed that after 48 h treatment, the MTT and liver injury index (ALT, AST, LDH) results showed that both 10 and 15 μM Sunitinib can cause cell death and significantly elevated liver enzymes, which meant both 10 and 15 μM Sunitinib can successfully build the hepatotoxicity model. Based on this, we further combined other small molecule inhibitors/agonist with 10 and 15 μM Sunitinib to explore the mechanism of hepatotoxicity. We seem to have overlooked the problem of concentration uniformity.

Autophagy and apoptosis often occur simultaneously, together with complex interactions, determining cell death or survival and the progression of disease (Wang, 2015). On the one hand, autophagy maintains cellular homeostasis by transporting intracellular damaged, denatured or aging proteins and organelles into lysosomes for digestion and degradation (Doria et al., 2013). Autophagy protected against dasatinb-induced hepatotoxicity by inhibiting apoptosis (Yang et al., 2015). On the other hand, excessive activation of autophagy could also result in cell death (Doria et al., 2013). Autophagy induced by gefitinib led to liver damage by promoting apoptosis (Luo et al., 2021). Zhao et al. also reported that autophagy contributes to Sunitinib-induced cardiotoxicity and claimed that inhibition of autophagy acted as an important strategy for alleviating Sunitinib-induced cardiotoxicity (Zhao et al., 2010). However, the occurrence and role of autophagy in Sunitinib-driven hepatotoxicity merits further investigation. In the current study, we found that when autophagy was inhibited, the survival rate of Sunitinib-treated cells was significantly decreased, the apoptosis-related protein PARP was significantly down-regulated and the cleaved-Caspase3 was significantly increased. This indicates that autophagy plays a protective role in Sunitinib-induced hepatotoxicity. We provided new insights into the relation between apoptosis and autophagy induced by Sunitinib. And the autophagy effect and molecular mechanisms of Sunitinib might differ among cells. One of the deficiencies in this study is that we only used the autophagy inhibitor CQ to investigate the effect of inhibiting autophagy on apoptosis and hepatotoxicity but did not use small interfering RNA (siRNA) to silence the essential autophagy related gene. Atg7^−/−^ mice will be considered in our future research.

Given that autophagy promotes tumor cell survival and induce drug resistance thereby (Corallo et al., 2020; Liu and Du, 2021). Sunitinib combined with CQ was considered to reduce pancreatic tumor growth through suppression of autophagy and increased apoptosis (Thakur et al., 2018). Furthermore, a variety of clinical trials have focused on the combination of CQ/HCQ and antitumor drugs (Wang et al., 2021). Of note, our findings suggested inhibiting autophagy blindly might lead to severe liver injury, subsequent drug discontinuance and exacerbation of cancer.

Our group previously observed that Sunitinib activated the oxidative stress, induced mitochondrial apoptosis and eventually lead to hepatotoxicity (Guo et al., 2021). Oxidative stress has been shown to be an important stimulus for hepatotoxicity (Cao et al., 2022; Jing et al., 2022). Studies have indicated that oxidative stress elicited by ROS excessive production of could result in apoptosis by activating MAPKs signaling pathways (Xue et al., 2012; Park et al., 2014). MAPKs signaling pathway is an important intracellular signal transduction system. Both JNK/MAPK and P38/MAPK are stress-induced activated protein kinases, which can be activated by various environmental stress factors, such as oxidative stress. JNK and p38/MAPK are known to be the upstream regulator of Bcl-2/Bax (Yue and Lopez, 2020), controlling apoptotic and survival processes (Cano and Mahadevan, 1995). In addition, Sorafenib induces apoptosis of EBV-transformed B cells through ROS-dependent JNK/p38-MAPK signaling in an ERK-independent manner (Park et al., 2014). This study was mainly focused on the role of JNK and P38 in Sunitinib-induced hepatotoxicity. Therefore, we may have overlooked the potential role of ERK.

Licorice, one of the most commonly prescribed Chinese herbal medicine, has been confirmed to possesses a number of biological activities, such as antioxidation and anti-inflammatory (Li et al., 2019). Clinically, glycyrrhiza preparations such as diammonium glycyrrhizin and magnesium isoglycyrrhizinate are commonly used in treating liver injury induced by various drugs, with the effect of “mediating all kinds of medicine and eliminating poison” (Wang et al., 2019; Wu et al., 2021a; Gong et al., 2022). GA is widely considered one of the main active substances of licorice and possesses a strong liver protective effect (Yang et al., 2017; Wu et al., 2021b). Li et al. (2022) found that magnesium isoglycyrrhizinate exerts therapeutic effects on crizotinib-induced hepatotoxicity by decreasing the level of ROS. Xu et al. (2022) also reported that GA significantly mitigated Sunitinib-induced cardiotoxicity. We have proved previously that GA could alleviate liver damage through upregulating Nrf2, defensing oxidative stress and inhibiting apoptosis (Cao et al., 2016; Yan et al., 2021). However, there are no reports on the effect of GA against Sunitinib-induced hepatotoxicity. Our study demonstrated for the first time the protective effects of GA against Sunitinib-evoked hepatotoxicity. The beneficial effects of GA might closely relate to the inhibition of apoptosis and its antioxidant activity. Sunitinib in combination with GA might be a promising therapeutic strategy in the future, reducing toxicity and increasing efficiency. Our present findings are preliminary and we will further validate the protective effect of GA on Sunitinib as well as the mechanism.

## Conclusion

**FIGURE 7:**
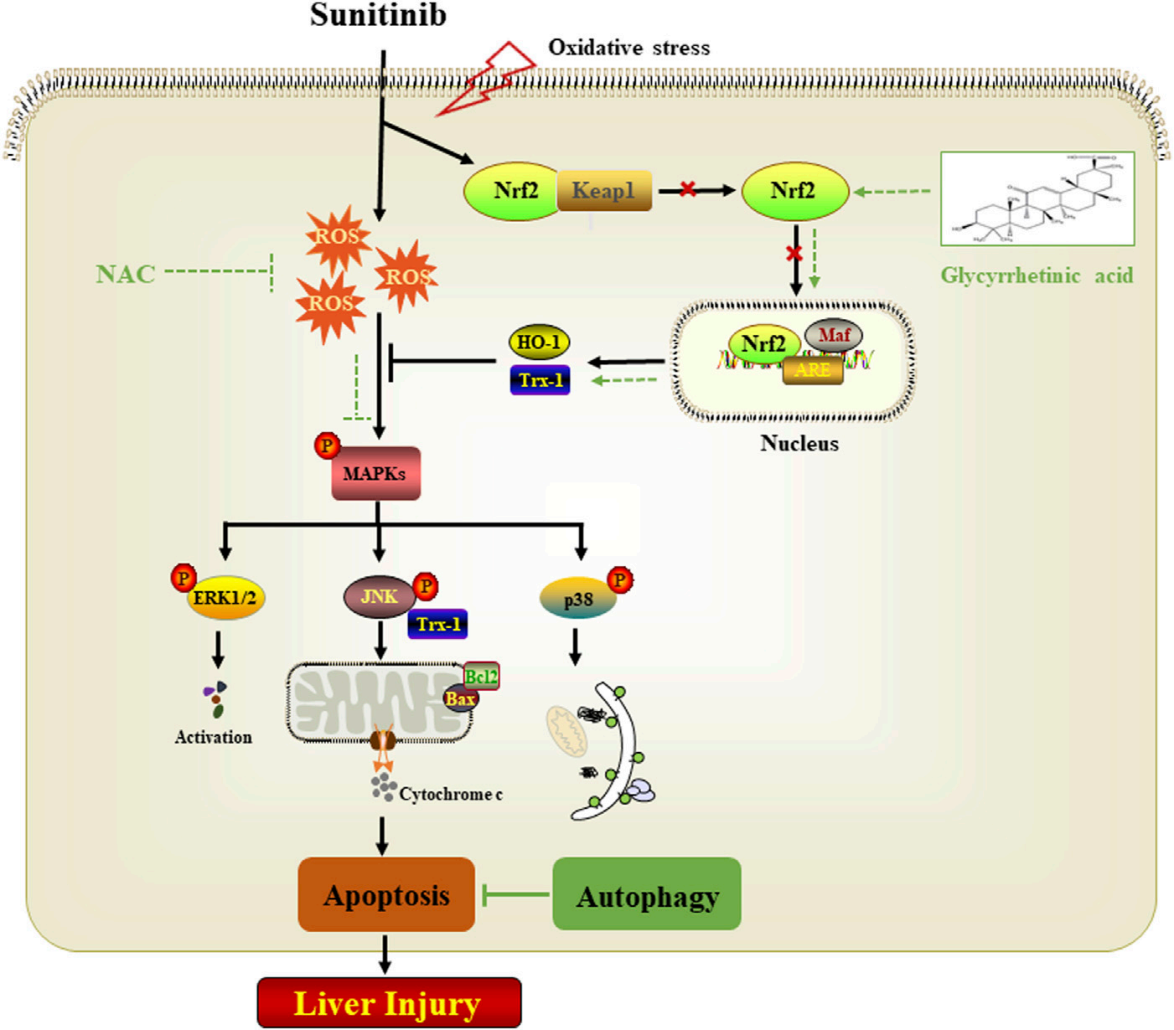
Proposed mechanism of Sunitinib-induced hepatotoxicity.

In conclusion, the present study provides novel evidence on the underlying mechanisms of Sunitinib-induced hepatotoxicity. We confirmed that apoptosis was mainly responsible for the hepatotoxicity of Sunitinib and revealed the adaptive protective role of autophagy. In terms of mechanism, ROS-mediated MAPKs pathway played a critical role in Sunitinib-induced cell damage. Furthermore, we also revealed that glycyrrhetinic acid may serve as a potential therapeutic agent against Sunitinib-induced liver injury by attenuating oxidative stress and apoptosis (Figure 7).

## Data Availability

The original contributions presented in the study are included in the article/Supplementary Material, further inquiries can be directed to the corresponding author.

## Abbreviations

ALT, alanine aminotransferase; AST, aspartate aminotransferase; LDH, lactate dehydrogenase; CQ, chloroquine; GA, glycyrrhetinic acid; MAPKs, mitogen-activated protein kinases; ERK, extracellular signal-regulated kinase; JNK, c-Jun N-terminal kinase; Nrf2, nuclear factor erythroid 2-related factor 2; ROS, reactive oxygen species; HO-1, heme oxygenase-1; NAC, N-Acety-L-Cysteine; 3-MA, 3-Methyladenine; c-Caspase3, cleaved-Caspase3.
