# Supplementary material for: Sunitinib induced hepatotoxicity in L02 cells via ROS-MAPKs signaling pathway
Source: Front Pharmacol. 2022 Oct 26;13:1002142. doi: 10.3389/fphar.2022.1002142 (PMC9643779; doi:10.3389/fphar.2022.1002142)
Supplement: Supplementary file 1 [file DataSheet1.docx]

***Supplementary Material***


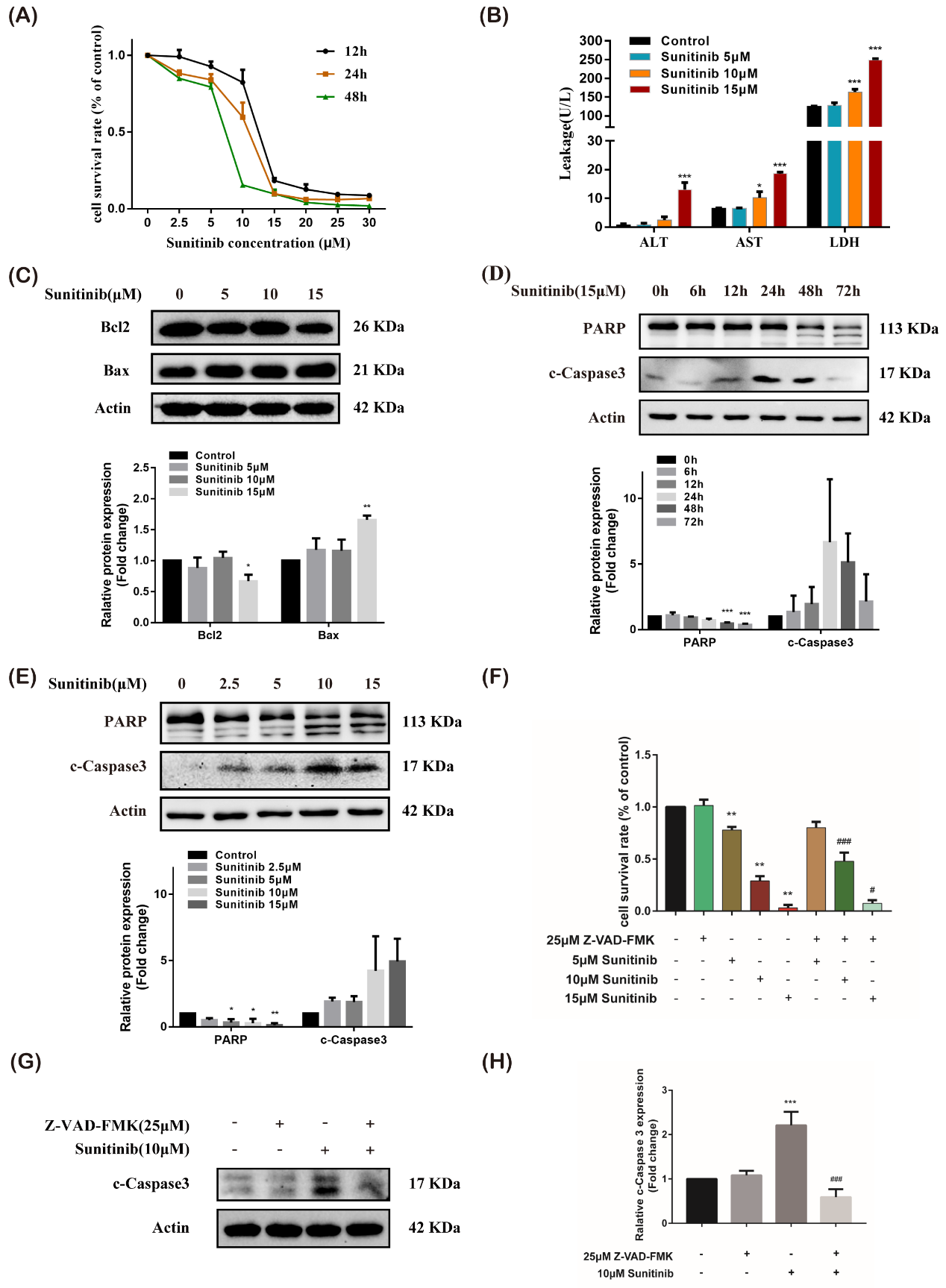


**Supplementary Figure 1.** Sunitinib damages L02 cells and induce apoptosis. (**A**) Cytotoxicity of Sunitinib alone at various concentration for 12, 24 and 48 h in L02 cells (n=4). (**B**) ALT, AST and LDH levels in the supernatant following Sunitinib exposure at 5, 10, 15μM for 48 h (n=3). (**C**)Bcl2 and Bax levels in L02 cells incubated with Sunitinib (0, 5, 10, 15 μM) for 48 h. (**D**) Western blot analysis for the level of PARP and c-Caspase3 after Sunitinib exposure (15μM) for 0, 6, 12, 24, 48, 72 h (n=3). (**E**) PARP and c-Caspase3 protein levels in L02 cells incubated with Sunitinib (0, 2.5, 5, 10, 15 μM) for 48 h (n=3). (**F**) The cell survival rate of LO2 cells after treatment with Sunitinib with or without Z-VAD-FMK (n=6). (**G,H**)Protein expression of c-Caspase3 after L02 cells were incubated with 10 μM Sunitinib for 48 h in the presence or absence of 25 μM Z-VAD-FMK pretreatment for 2 h (n=3). ^*^P<0.05, ^**^P<0.01 and ^***^P<0.001 vs. Control group. ^#^P<0.05 and ^###^P<0.001 vs. Sunitinib group.


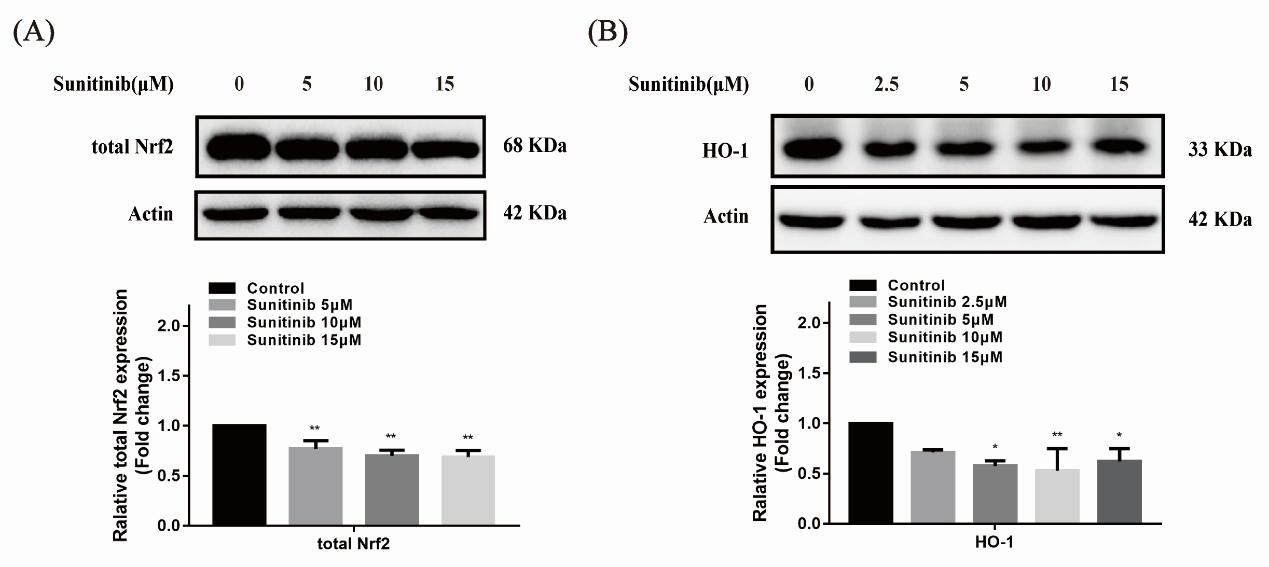


**Supplementary Figure 2.** Sunitinib downregulates the protein expression of Nrf2 and HO-1. (**A,B**)Total Nrf2 and HO-1 levels in L02 cells after Sunitinib exposure at different concentrations for 48 h (n=3). ^*^P<0.05, ^**^P<0.01 vs. Control group.


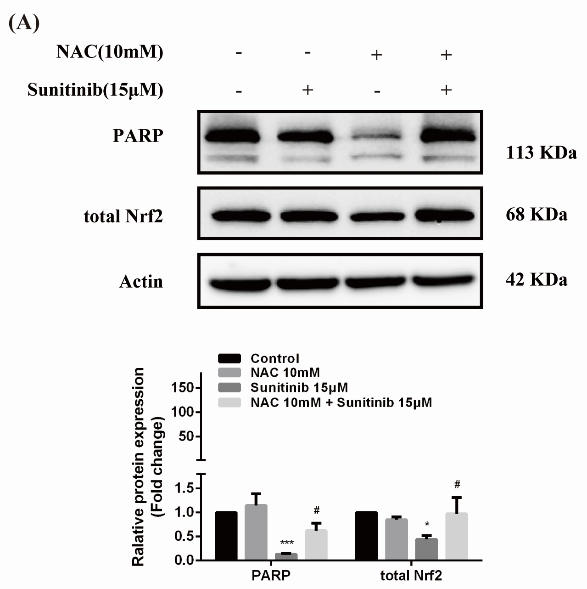


**Supplementary Figure 3.** NAC reversed the expression of PARP and Nrf2 downregulated by Sunitinib. **(A)**Total Nrf2, PARP in L02 cells after Sunitinib (15 μM) for 48 h in the presence or absence of 10 mM NAC pretreatment for 6 h (n=3). ^*^P<0.05 and ^***^P<0.001 vs. Control group. ^#^P<0.05 vs. Sunitinib group.
